# Large-scale expansion of human umbilical cord-derived mesenchymal stem cells using PLGA@PLL scaffold

**DOI:** 10.1186/s40643-023-00635-6

**Published:** 2023-03-08

**Authors:** Yujie Liu, Obed Boadi Amissah, Xiaoying Huangfang, Ling Wang, Jean de Dieu Habimana, Linshuang Lv, Xuanyan Ding, Junyi Li, Ming Chen, Jinmin Zhu, Omar Mukama, Yirong Sun, Zhiyuan Li, Rongqi Huang

**Affiliations:** 1 CAS Key Laboratory of Regenerative Biology, Guangdong Provincial Key Laboratory of Stem Cell and Regenerative Medicine, Guangzhou Institutes of Biomedicine and Health, Chinese Academy of Sciences, Guangzhou, 510530 China; 2 Guangzhou Junyuankang Biotechnology Co., Ltd., Guangzhou, 510530 China; 3 University of Chinese Academy of Sciences, Beijing, 100049 China; 4 School of Medicine, Guilin Medical University, Guilin, 541199 China; 5 School of Life Sciences, University of Science and Technology of China, Hefei, 230027 China; 6 GZMU-GIBH Joint School of Life Sciences, Guangzhou Medical University, Guangzhou, 511436 China; 7 GIBH-HKU Guangdong-Hong Kong Stem Cell and Regenerative Medicine Research Centre, Guangzhou Institutes of Biomedicine and Health, Chinese Academy of Sciences, Guangzhou, 510530 China; 8 Department of Anatomy and Neurobiology, Xiangya School of Medicine, Central South University, Changsha, 410013 China

**Keywords:** MSCs, Large-scale expansion culture, PLGA@PLL scaffold, Biomedicine

## Abstract

Mesenchymal stem cells (MSCs) are highly important in biomedicine and hold great potential in clinical treatment for various diseases. In recent years, the capabilities of MSCs have been under extensive investigation for practical application. Regarding therapy, the efficacy usually depends on the amount of MSCs. Nevertheless, the yield of MSCs is still limited due to the traditional cultural methods. Herein, we proposed a three-dimensional (3D) scaffold prepared using poly lactic-co-glycolic acid (PLGA) nanofiber with polylysine (PLL) grafting, to promote the growth and proliferation of MSCs derived from the human umbilical cord (hUC-MSCs). We found that the inoculated hUC-MSCs adhered efficiently to the PLGA scaffold with good affinity, fast growth rate, and good multipotency. The harvested cells were ideally distributed on the scaffold and we were able to gain a larger yield than the traditional culturing methods under the same condition. Thus, our cell seeding with a 3D scaffold could serve as a promising strategy for cell proliferation in the large-scale production of MSCs. Moreover, the simplicity and low preparation cost allow this 3D scaffold to extend its potential application beyond cell culture.

**Graphical Abstract:**

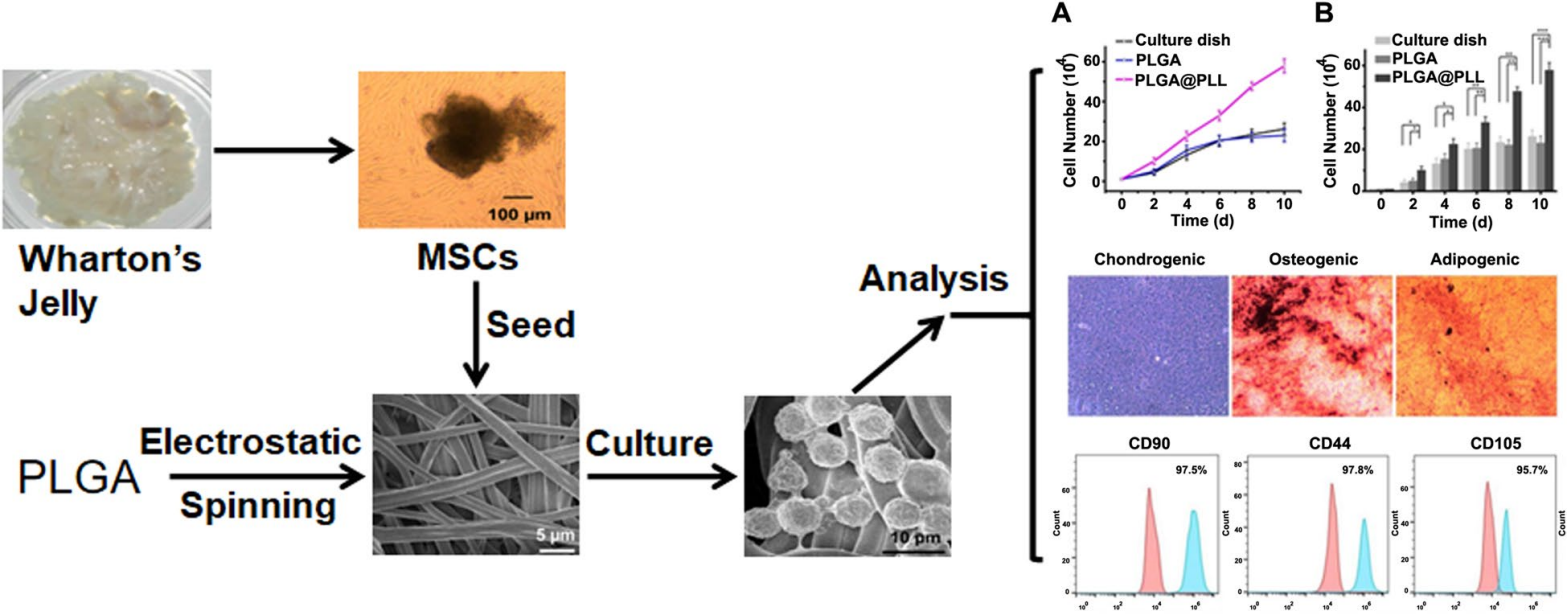

**Supplementary Information:**

The online version contains supplementary material available at 10.1186/s40643-023-00635-6.

## Introduction

In the past decades, mesenchymal stem cells (MSCs) have been extensively investigated. Thus far, MSCs have been applied to clinical trials for treatment (Lan et al. 2021). At present, MSCs could be isolated from diverse tissues and organs, including bone marrow, placenta, umbilical cord, hair follicles, fat, and body fluids (Huang et al. 2009; Lin et al. 2019; Ryu et al. 2014). Among these sources, the umbilical cord is not only easily achieved but also relatively convenient in the harvest of MSC.

The possibility of non-invasive harvesting and abundant source, no ethical issues, and low immunogenicity of HUC-MSCs give them a unique advantage in clinical applications. In recent years, HUC-MSCs have been widely used in treatment of multiple types of disease and have achieved good therapeutic effects (Xie et al. 2020; Yin et al. 2019; Li et al. 2005).

MSCs therapy based on hUC-MSCs is usually utilized to restore physiological function for the damaged parts of the body. The damage may be from injury, aging, or even complications of previous surgery (Caplan et al. 2011; Xu et al. 2018). Regardless of the different reasons, hUC-MSCs can contribute to significant pain relief and reverse the damage caused by aging or other external factors. The therapy works by the harvested hUC-MSCs and then injecting them into the afflicted area. When MSCs are applied to a specific area, they could release growth factors that promote healthy cells to proliferate (Lu et al. 2019; Lin et al. 2012). As stem cells, they can transform into various tissues or structures, including bone, cartilage, fibrous tissues such as muscles and ligaments, and even nerve tissue (Reboredo et al. 2016; Zhou et al. 2017; Pumberger et al. 2016). MSCs therapy holds great potentials for the cure of various types of disease, including COVID-19 (Zhu et al. 2021; Shu et al. 2020; Lanzoni et al. 2021).

MSC therapy usually depends not only on its native characteristics, such as self-renewal activity, genomic stability, and differentiation capability, but also largely on its large-scale production (Ying et al. 2008; Weiss et al. 2019; Jiang et al. 2002). Although MSCs are attractive in clinical applications, their yield and native properties are still limited to the traditional mechanical culture substrates, including culture flask or culture dish, which yields a small number of cells in a short time (Ng et al. 2021; Han et al. 2012). To obtain MSCs in great quantities, these culture strategies usually depend on expanding the bottom surface of the substrate and continuous passage culture of cells. As a result, the traditional methods easily lead to cell senescence and loss of multipotency (Zhao et al. 2015; Li et al. 2005). In addition, these methods also have a strong contact inhibition effect on cell proliferation. That is, the high density of cells could inhibit cell division and growth when confluence reaches 100% (Kim et al. 2016). Therefore, a high-performance approach to MSC (including hUC-MSCs) culture is greatly required.

Electrospinning is a technique that utilizes polymer solutions and strong electric fields to produce nanosized materials that have wide-ranging applications (Stojanov et al. 2020). To date, electrospinning technology has produced a series of substrates, such as organic, organic–inorganic composite, and inorganic nanofibers (Li et al. 2004; Greiner et al. 2007; Yu et al. 2017). These materials possess several unique properties, including excellent biocompatibility, large surface areas, and good porosity (Go et al. 2016; Gong et al. 2020). The development and improvement of electrospinning require synthesizing a more advanced material that could perform well with a controllable process, low cost, and convenient steps. Moreover, they have been applied to various fields, such as biomedicine, catalysis, energy, optoelectronics, and food engineering (Sill et al. 2008; Liu et al. 2018; Lu et al. 2009).

By employing these benefits, we developed a poly (lactic-co-glycolic acid) (PLGA) nanofiber scaffold grafted with polylysine (PLGA@PLL) for large production of umbilical cord-derived mesenchymal stem cells (hUC-MSCs). The PLGA@PLL scaffolds possess unique physical and chemical properties, such as good biocompatibility, stability, and cyclic utilization, which are beneficial to cell culture. Thus, the cells were enabled to realize multi-directional and multi-level amplification on this three-dimensional (3D) scaffold.

In this study, we synthesized a PLL-grafted PLGA nanofibrous scaffold by electrospinning technology. We found that the PLGA@PLL scaffold exhibited a better affinity toward hUC-MSCs than an unmodified one. Application of this scaffold to hUC-MSCs culture gained a higher cell proliferation rate, less senescent than those in the traditional culture dish. Further cellular analysis revealed the morphology, the original properties and the multipotency of the hUC-MSCs harvested from the PLGA@PLL scaffold are comparable to the native MSCs. These advantages together with the simplicity, inexpensiveness, and the feasibility of preparation of the scaffold rendered it an attractive substrate for the in vitro production of hUC-MSCs at a large scale.

## Materials and methods

### Materials

The carboxyl-terminated poly lactic-co-glycolic acid (PLGA) was purchased from Meilunbio (Dalian, China). Polylysine (PLL), 4-dimethylaminopyridine (DMAP), and 1-ethyl-3-(3-dimethylamino-propyl) carbodiimide (EDC) were all purchased from Sigma-Aldrich (Guangzhou, China). Cell counting kit-8 (CCK-8) and senescence β-galactosidase staining kit were obtained from Beyotime Biotechnology (Shanghai, China). Human stem cell pluripotency detection kit was purchased from ScienCell (Shanghai, China). A human MSC analysis kit (562245) for flow cytometry assay was obtained from BD Biosciences (Shanghai, China). The reagents for real-time PCR were obtained from Vazyme Biotech Co., Ltd (Nanjing, China). DMEM/F12 medium was purchased from Weijia Biotechnology (Guangzhou, China).

### Instrumentation

The PLGA@PLL nanofiber scaffold was prepared by an electrospinning machine (TEADFS-103). The PLGA@PLL scaffolds before and after cell seeding were imaged by Scanning electron microscopy (SEM, Phenom) with an acceleration voltage of 30 kV (JSM-7500F). The absorbance of hUC-MSCs in the CCK-8 kit assay was detected by a microplate reader (Bio-Tek Instrument, Winooski). The hUC-MSCs after β-galactosidase staining were observed by a fluorescence microscope (Leica). The protein expression was identified using flow cytometry (CyFlow Space).

### Preparation of PLGA@PLL scaffold

The PLGA scaffold was made according to previously described methods (Zhao et al. 2008; Minardi et al. 2016). Briefly, PLGA was dissolved in a mixture solution of tetrahydrofuran and dimethylformamide (THF: DMF = 70: 30), with a final concentration of 18%. Then the resultant solution was loaded in a 10 mL syringe equipped with an 18-mm-inner diameter needle. Afterward, the mixture system was subjected to electrospinning at 16 kV voltage, 4 mL/h push rate, and 15 cm needle-collector distance, under 22 °C and 45% humidity. The collector was rotated at 200 rpm, and after 10 h, the PLGA nanofiber was collected and washed three times with ddH_2_O for further use. To enhance cell adhesion toward the PLGA scaffold, a sterile carboxyl-terminated PLGA nanofiber was grafted with PLL (80 ng/mL) with crosslinking reaction via 1% EDC and DMAP (2 mg) for 24 h at room temperature. Next, the scaffold was washed three times with ddH_2_O prior to cell seeding.

### Characterization of PLGA@PLL scaffold by SEM

The morphology of the PLGA@PLL scaffolds before and after cell seeding was characterized by a scanning electron microscope (SEM, Phenom). The cells seeded on the scaffold were fixed with 4% paraformaldehyde, washed with PBS, and left to dry. All samples were fixed, sputtered with gold, and imaged using SEM at an accelerating voltage of 5 kV. To estimate the porosity and pore size of the PLGA@PLL scaffolds, SEM images of the scaffolds were analyzed by the ImageJ software.

### Degradation of PLGA and PLGA@PLL scaffolds

For degradation tests, scaffolds (*n* = 5) were incubated for 4 weeks in DMEM medium at 37 °C with 5% CO_2_ in an incubator. At each time point (Weeks 1, 2, 3, and 4), the samples were removed from the DMEM medium, vacuum dried (70 °C, 6 h), and weighed on an analytical balance with a sensitivity of 0.1 mg, and the change in weight (%) was calculated. The pH value of the DMEM medium was also measured at the end of each time point, and the change in pH value with respect to time was plotted.

### Isolation of MSCs from human umbilical cord

The umbilical cords and bone marrow-derived mesenchymal stem cells (BM-MSCs) were obtained from healthy volunteers (Guangzhou Red Cross Hospital) in accordance with relevant laws and approval from the Guangzhou Institutes of Biomedicine and Health Ethics Committee. Briefly, MSCs were isolated from the umbilical cord as follows. Firstly, the umbilical cord was washed thoroughly with PBS containing 1% penicillin–streptomycin and then cut into pieces. Subsequently, blood vessels and the residual blood were removed, resulting in Wharton’s jelly. Next, the Wharton’s jelly was sliced into small pieces of 1–3 mm with ophthalmic scissors, suspended in PBS and then centrifuged at 2000 rpm for 3 min to collect the precipitate. In a sterile culture dish, DMEM medium supplemented with 10% FBS and 1% penicillin–streptomycin was added. Subsequently, the Wharton’s jelly was added to the medium and incubated at 37 °C with 5% CO_2_. After a while, the hUC-MSCs appeared as a colony and were used for subsequent experiments. BM-MSCs were maintained in our lab by a similar culturing method.

### hUC-MSCs culture on PLGA@PLL scaffold

The sterilized scaffolds were cut into squares with an area of 1 cm × 1 cm. Before cell seeding, the scaffolds were immersed in DMEM medium in a 6-well plate. Then, hUC-MSCs (1 × 10^5^ cells/mL, 100 μL) were, respectively, seeded on a traditional culture dish, PLGA scaffold, and PLGA@PLL scaffold for different times ranging from 2 to 10 days, under 37 °C with 5% CO_2_. Each group was repeated at least six times. Once the cells were harvested, cell proliferation was estimated by cell counting and CCK-8 assay.

### Real-time qPCR analysis

Total RNA derived from hUC-MSCs seeded on the PLGA@PLL scaffold or culture dish was isolated by TRIzol Reagent (Tiangen, Beijing, China) according to the manufacturer’s instructions. The samples were reverse-transcribed and subjected to real-time PCR using the HiScript III RT SuperMix (Vazyme, Nanjing, China) for reverse-transcribed PCR and Taq Pro Universal SYBR qPCR Master Mix (Vazyme, Nanjing, China) for qPCR. Beta-actin was used as an internal control for normalization. Primer sequences used in this study are listed in Additional file 1: Table S1. Results were analyzed using the 2^−△△Ct^ method.

### β-Galactosidase staining analysis

The hUC-MSCs from the PLL-PLGA scaffold with a density of 1 × 10^5^ cells/well (100 μL) were seeded for β-Galactosidase staining analysis. As well, native hUC-MSCs with the same density from Wharton’s jelly were used as a control. Briefly, the hUC-MSCs of two groups were left to be serially cultured in an incubator at 37 °C with 5% CO_2_. After 10 days, the cells from different groups were collected and then, respectively, cultured in a 6-well plate with the same density (2 × 10^4^ cells/well, 500 μL). When confluence reached 100%, the hUC-MSCs in each group were treated with a β-galactosidase kit according to the manufacturer’s instructions. After that, all samples were observed by a fluorescence microscope to assess the cell senescence.

### Multipotency assay

To study whether the hUC-MSCs from the PLGA@PLL scaffold had the possibility to be induced into other cell types, a multipotency test was performed. Native hUC-MSCs from Wharton’s jelly and BM-MSCs derived from the PLGA@PLL scaffold were used as controls. A multipotency assay was conducted using commercial kits as follows. To obtain chondroblasts, all groups were treated with a chondrogenic medium containing DMEM, 10% FBS, insulin (6.25 μg/mL), transforming growth factor-beta 1 (10 ng/mL), and ascorbate-2-phosphate (50 nM). To obtain osteoblasts, all groups were cultured in an osteogenic medium containing DMEM, 10% FBS, β-glycerophosphate (10 mM), dexamethasone (0.1 μM), and ascorbate-2-phosphate (50 μM). To obtain adipoblasts, all groups were treated with adipogenic medium containing DMEM, 10% FBS, dexamethasone (1 μM), isobutylmethylxanthine (0.5 mM), indomethacin (200 μM), and insulin (10 μM). For each group, the medium was replaced every three days. After chondrogenic, osteogenic, or adipogenic induction, samples were stained with toluidine blue, Alizarin red, or oil red O, respectively. Native hUC-MSCs from Wharton’s jelly were used as a control.

### Flow cytometry analysis of hUC-MSCs

The expression levels of hUC-MSCs surface markers were determined with flow cytometry. Native hUC-MSCs obtained from Wharton’s jelly were used as a control. Briefly, 1 × 10^4^ native hUC-MSCs isolated from Wharton’s jelly and 1 × 10^4^ hUC-MSCs released from PLGA scaffolds were added in a mixture of 0.2% Triton-X and 3% BSA for permeabilization and blocking, respectively. Afterward, the hUC-MSCs of both two groups were similarly incubated with FITC mouse anti-human CD90, PE mouse anti-human CD44, PerCP/Cy5.5 anti-human CD105, and APC mouse anti-human CD73. After that, all the hUC-MSCs were incubated with Alexa Fluor 488-conjugated anti-mouse at room temperature for 1 h in the dark. Subsequently, the samples were subjected to flow cytometry (AccuriC6, BD Biosciences) and analyzed with FlowJo software.

### Statistical analysis

Unless noted otherwise, data are presented as mean ± SEM of at least triplicate samples for different groups. In each case, the differences between each group were performed using a paired *t*-test or a one-way ANOVA test. *p* < 0.05 or < 0.01 denoted a significant difference between the two groups.

## Results

### Characterization and stability of PLGA@PLL scaffold

After preparation, the microarchitecture of PLGA@PLL scaffold was characterized by a scanning electron microscope (SEM). We found that pore sizes within these scaffolds ranged from 2.1 μm to 16.9 μm; over 73% of the pore sizes ranged from 5 μm to 11 μm and the whole scaffold possessed an average pore size of 8.6 ± 3.1 μm (Additional file 1: Fig. S1), which is easily permeated with culture medium and to effectively accommodate incorporation of hUC-MSCs into the scaffold and facilitate the hUC-MSCs to grow.

Moreover, degradation tests revealed the PLGA@PLL scaffold hardly degraded in the culture medium during a period of four weeks **(**Additional file 1: Fig. S2A, B) and the pH value of the culture medium remained at around 7.4 in this period (Additional file 1: Fig. S2C). Taken together, the appropriate pore size range and stable physicochemical properties of the PLGA@PLL scaffold make it a suitable, sustainable substrate for hUC-MSCs culture in vitro.

### hUC-MSCs seeding on the PLGA@PLL scaffold

**Fig. 1:**
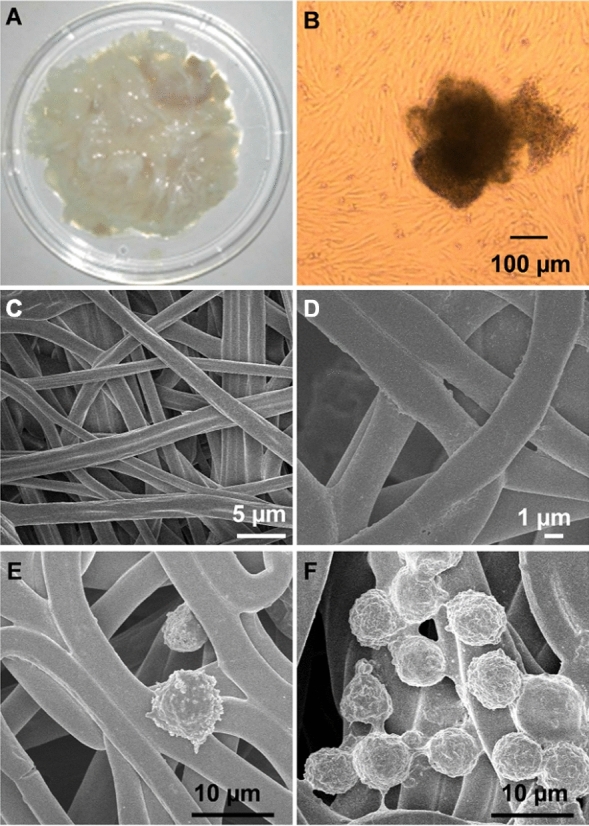
Cell seeding on the PLGA@PLL scaffold. **A** Wharton’s jelly was harvested in pieces from the human umbilical cord. **B** MSCs were cultured from Wharton’s jelly tissues and displayed a spindle-like shape. **C** SEM image of PLGA@PLL scaffold prepared using electrospinning technique. **D** A magnified SEM image of PLGA@PLL scaffold with a fibrous structure. **E** hUC-MSCs were incubated on the unmodified PLGA scaffold. **F** hUC-MSCs were incubated on the PLGA@PLL scaffold

The umbilical cord was obtained with the consent of the volunteers. According to the above method, Wharton’s jelly was easily and successfully isolated from the umbilical cord using this approach. Then, the Wharton’s jelly was separated into pieces **(**Fig. 1A**)**. After Wharton’s jelly was incubated in an incubator for 3 weeks, the hUC-MSCs grew against the wall of the culture dish, formed a monolayer, and presented fibroblast-like shape (Fig. 1B and Additional file 1: Fig. S3A). The newly prepared PLGA scaffold displayed a three-dimensional fibrous structure. After PLL modification, the resultant scaffold (PLGA@PLL) remained in the original structure (Additional file 1: Fig. S4). Subsequently, the PLGA@PLL scaffold was characterized by SEM and exhibited a uniform fiber construction (Fig. 1C). Furthermore, to observe the microstructure of this scaffold, a magnified SEM image was collected and revealed that the diameter of the scaffold was approximately 3–4 μm (Fig. 1D).

For cell seeding, the hUC-MSCs could spontaneously absorb on the scaffold owing to the positive charge of PLL. Meanwhile, an unmodified PLGA scaffold with cell seeding served as a control. As shown in Fig. 1E, the hUC-MSCs on the scaffold exhibited spherical morphology under SEM and had an average size of 8 μm. Moreover, hUC-MSCs with higher density distributed on the PLGA@PLL scaffold than the control one (Fig. 1F and Additional file 1: Fig. S5), indicating that the PLGA@PLL scaffold had an advantage over the PLGA scaffold for cell culture.

### Evaluation of cell proliferation

**Fig. 2:**
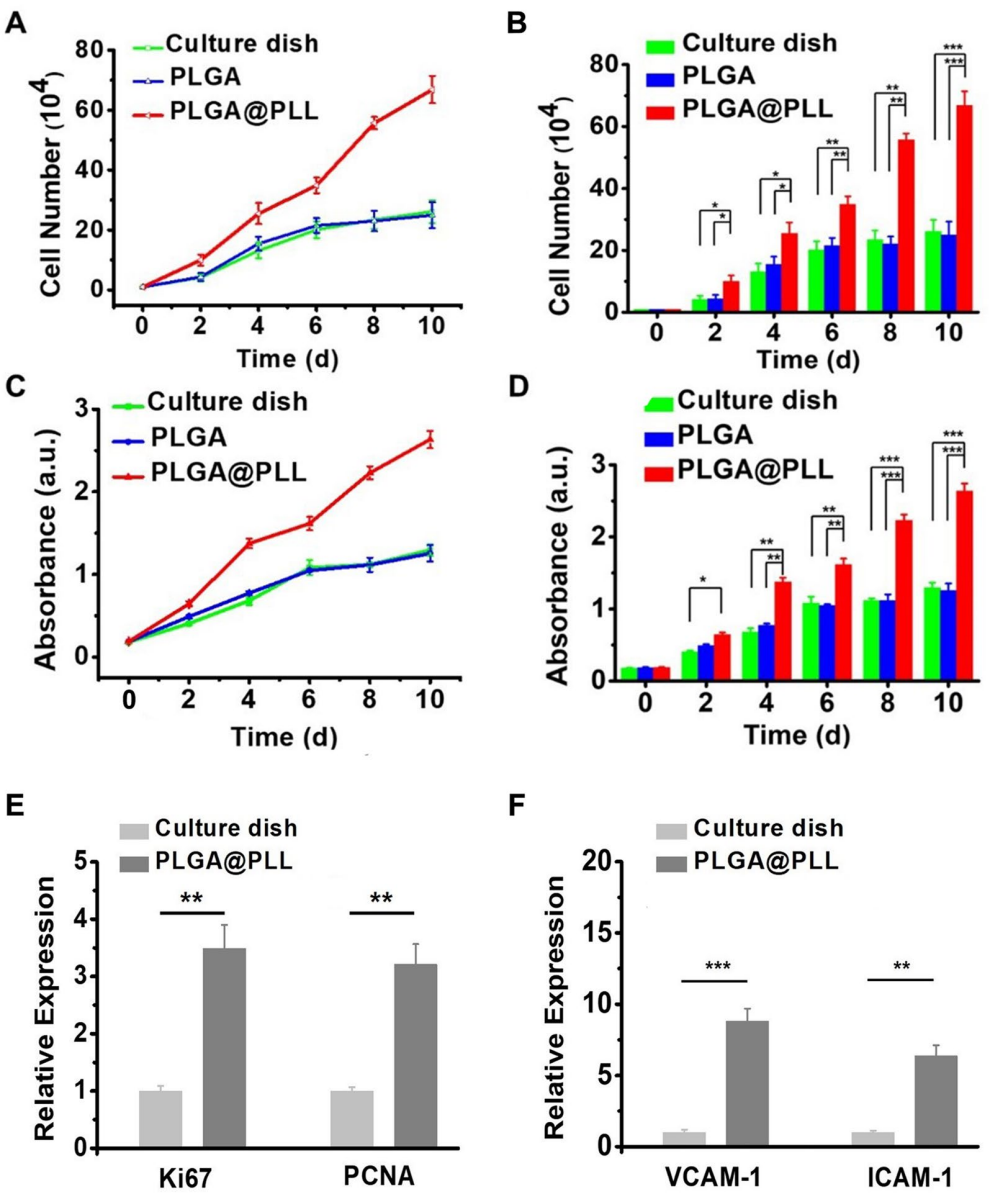
Cell proliferation was evaluated by cell counting and CCK-8 assay. **A** Cell growth curves of three groups, including culture dish, PLGA scaffold, and PLGA@PLL scaffold, were made according to cell counting. **B** Statistical analysis of cell proliferation according to CCK-8 assay (*n* = 6). **C** Cell growth curves of hUC-MSCs seeded on the culture dish, PLGA scaffold, and PLGA@PLL scaffold were made based on cell counting. **D** Statistical analysis of cell proliferation based on CCK-8 assay (*n* = 6). **E** Expression levels of the Ki67 and PCNA genes among the control and the scaffold groups were compared (*n* = 5). **F** Expression levels of the VCAM-1 and ICAM-1 genes among the control and the scaffold groups were compared (*n* = 5). The data were normalized to the internal control β-actin and are plotted. *indicates *p* < 0.05, **indicates *p* < 0.01, ***indicates *p* < 0.001, two-tailed t-test

To explore the feasibility of hUC-MSCs culture on the new-synthesized scaffold, the hUC-MSCs were cultured and harvested on a culture dish, PLGA, and PLGA@PLL scaffolds, respectively, for different periods, from 0 to 10 days. Next, cell proliferation was performed with cell counting. In Fig. 2A, the cell proliferation curves indicated that hUC-MSCs grew faster and generated a high yield seeded on the PLGA@PLL scaffold compared to the culture dish and PLGA scaffold. As indicated in a bar graph (Fig. 2B), on the 4th day, the number of hUC-MSCs seeded on the PLGA@PLL scaffold increased to 25.51 × 10^4^, that of the culture dish group increased to 13.17 × 10^4^, and that of the PLGA scaffold group increased to 15.47 × 10^4^. Cell proliferation for the PLGA@PLL group displayed a great difference compared to the latter two groups (*p* < 0.01; two-tailed *t*-test). It showed a similar trend after 10 days of cell culture (*p* < 0.001; two-tailed *t*-test). Therefore, this assay revealed that the PLGA@PLL scaffold facilitated cell growth and proliferation, leading to a large production of MSCs.

To further verify the superior performance of the PLGA@PLL scaffold in cell culture, we also made the cell proliferation curves similarly taken by the CCK-8 assay. The data revealed that hUC-MSCs exhibited faster growth potential on the PLGA@PLL scaffold than that of the culture dish and PLGA scaffold (Fig. 2C). As shown in Fig. 2D, on the 4th day, the absorbance of PLGA@PLL group was up to 1.38, and that of culture dish group and PLGA group was increased to 0.68 and 0.77, respectively. The result demonstrated that the PLGA@PLL group exhibited a greater difference than the culture dish group (*p* < 0.01; two-tailed *t*-test). The trend remained the same after the 8th day of culture (*p* < 0.001; two-tailed *t*-test). These findings further corroborated that the PLGA@PLL scaffold was a good substrate that could promote cell growth rapidly.

To explore the underlying mechanism of the prolific phenotype of hUC-MSCs derived from the PLGA@PLL scaffold, we further conducted a real-time qPCR in respective hUC-MSCs to analyze the expression of genes which encode for the key cellular proliferation biomarkers (Ki67 and PCNA, Huang et al. 2009; Lim et al. 2020) and the cellular adhesion biomarkers (VCAM-1 and ICAM-1, Furuta et al. 2021; Scott et al. 2013). The data shown that the expression level of all these genes were markedly higher in hUC-MSCs harvested from the PLGA@PLL scaffold compared to those from the culture dish and PLGA scaffold (Fig. 2E, F. PLGA@PLL scaffold vs. culture dish, Ki67, *p* < 0.01; PCNA, *p* < 0.01; VCAM-1, *p* < 0.001; ICAM-1, *p* < 0.01; all analyzed by two-tailed *t*-test). These results suggested that the faster proliferation of hUC-MSCs in the PLGA@PLL scaffold may attribute to the higher expression levels of cellular proliferation and adhesion genes in cells cultured on the scaffold. Therefore, the PLGA@PLL scaffold could serve as an ideal substrate for the hUC-MSCs’ growth and obtain high yields.

### Estimation of cell senescence by β-Galactosidase staining analysis

**Fig. 3:**
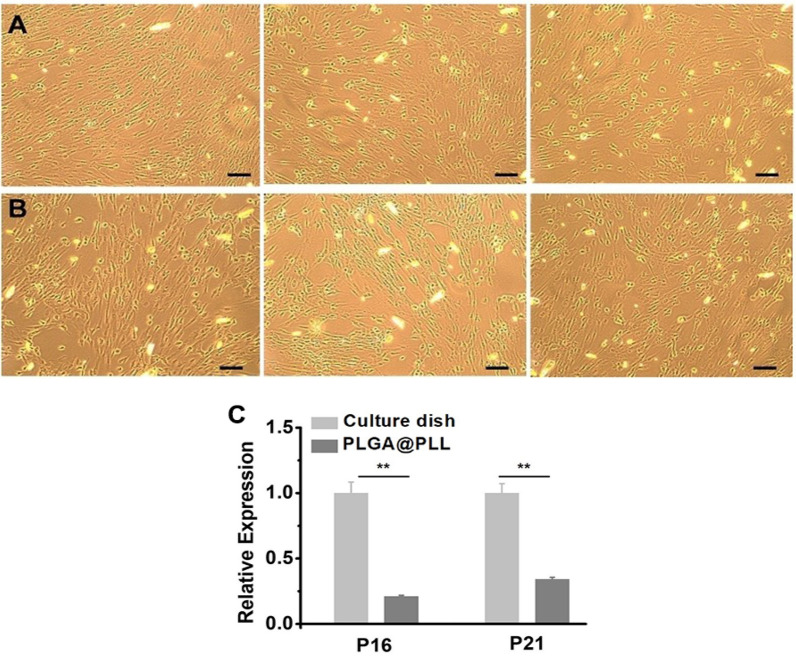
Cell senescence was analyzed by β-galactosidase staining and RT-qPCR. **A** native hUC-MSCs from Wharton’s jelly and **B** hUC-MSCs released from PLGA@PLL scaffold. After β-galactosidase staining, all samples were observed under a microscope and displayed in three random regions. **C** Expression levels of the key cellular senescence genes P16 and P21 among the control and the scaffold groups were compared. The real-time PCR data were normalized to the internal control β-actin and plotted (*n* = 5). **indicates *p* < 0.01, two-tailed *t*-test Scale bar: 100 μm

To assess the senescence of hUC-MSCs seeded on the PLGA@PLL scaffold, we conducted a β-galactosidase staining analysis. Herein, native hUC-MSCs from Wharton’s jelly and hUC-MSCs released from the PLGA@PLL scaffold were, respectively, incubated with a β-galactosidase staining kit. The result showed no cells with blue color (marked senescent cells) for both two groups. That is, the hUC-MSCs from each group maintained their good state and did not senesce (Fig. 3A, B). In addition, there was no difference in cell senescence for all groups. Therefore, the cell culture system for the PLGA@PLL scaffold verified no adverse effect on hUC-MSCs growth and property. To further explore cell aging of hUC-MSCs derived from the PLGA@PLL scaffold, the expression of two key cellular senescence genes, including P16 and P21, were determined by a real-time qPCR assay. These markers were expressed at levels sufficient to establish and maintain age-related growth retardation; they were usually used to identify senescent cells in tissues and cultured cells (Dodig et al. 2019). Specifically, the high expression of p16 would promote cell aging. The results shown that cells obtained from the PLGA@PLL scaffold expressed lower levels of P16 and P21 than that of the culture dish **(**Fig. 3C, p < 0.01, two-tailed *t*-test**)**, indicating that the hUC-MSCs seeded on the PLGA@PLL scaffold were capable of keeping a “young” state.

### Multipotency determination of hUC-MSCs

**Fig. 4:**
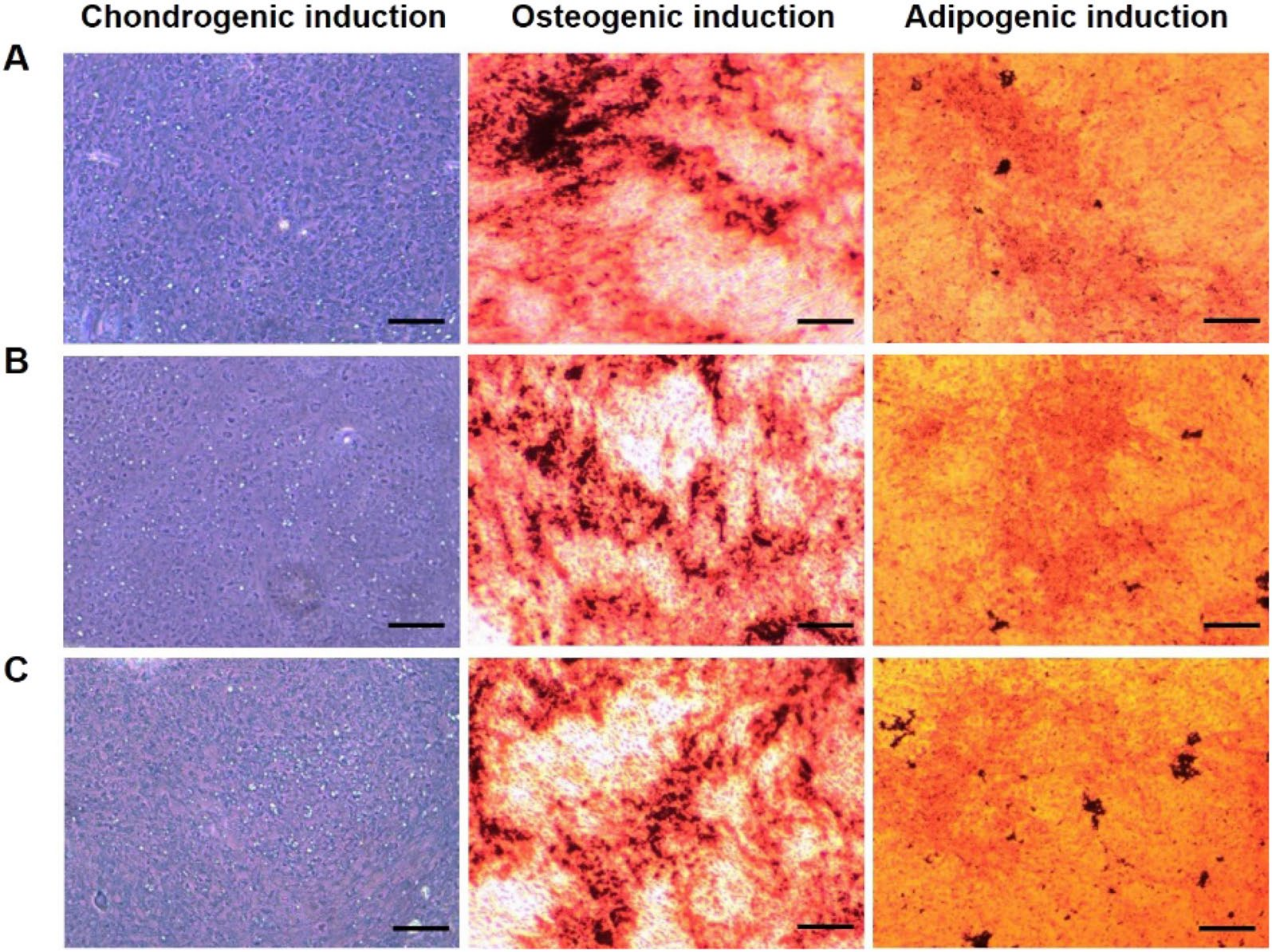
Determination of the multipotency for hUC-MSCs. **A** Native MSCs from Wharton’s jelly, **B** hUC-MSCs, and **C** BM-MSCs released from the PLGA@PLL scaffold were incubated with a multipotency detection kit and then were induced to differentiate into chondroblasts stained with toluidine blue, osteoblasts stained with Oil red O, and adipoblast stained with alizarin red. Scale bar: 100 μm

It has been reported that hUC-MSCs can be induced to differentiate into diverse kinds of cells in vitro. A multipotency assay was performed to determine if hUC-MSCs released from the PLGA@PLL scaffold maintained their property. Briefly, the hUC-MSCs were harvested from the scaffold and then incubated in different culture systems, including chondrogenic, osteogenic, and osteogenic media, for a period. The native MSCs (cells isolated from Wharton’s jelly) served as a control. To further verify the multipotency-supportive performance of the PLGA@PLL scaffold, bone marrow-derived mesenchymal stem cells (BM-MSCs) released from the scaffold were also examined with regard to their multipotency. After the abovementioned incubation, each group’s resultant products were stained with corresponding dyes. As illustrated in Fig. 4, native MSCs (Fig. 4A), the released hUC-MSCs (Fig. 4B), and the released BM-MSCs (Fig. 4C) were all successfully differentiated into chondroblasts, osteoblasts, and adipoblasts. The morphological properties and quantities of these differentiated tissues were comparable among the 3 groups. Taken together, the results indicated that the hUC-MSCs harvested from the PLGA@PLL scaffold still maintained their multipotency which can be differentiated into multiple cells and tissues and be further used for downstream trials.

### Biomarkers determination of hUC-MSCs

**Fig. 5:**
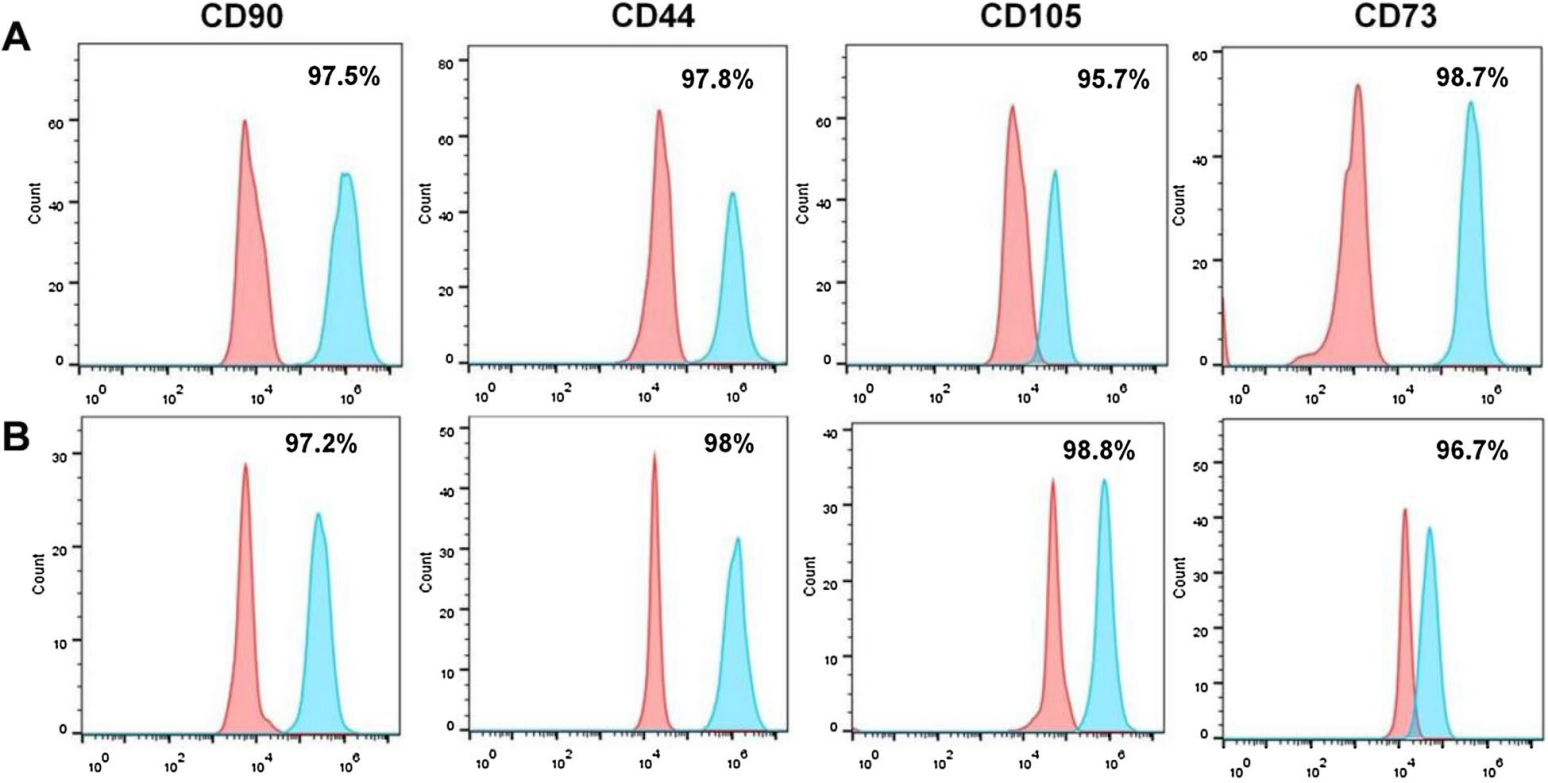
Specific hUC-MSCs markers were identified by flow cytometry. **A** Markers (CD90, CD44, CD105, and CD73) of the released hUC-MSCs from the PLGA@PLL scaffold. **B** The native MSCs from Wharton’s jelly served as controls

To further determine the multipotency of released hUC-MSCs from the PLGA@PLL scaffold and whether they had changed or otherwise, expression levels of their surface marker were evaluated by fluorescence-activated cell sorting (FACS). In parallel, the native hUC-MSCs were used as a control. The result showed that the released cells expressed high levels of proteins, including CD44, CD73, CD90, and CD105, which were the main cell surface markers of hUC-MSCs (Fig. 5A). This showed a similar result to that of the native hUC-MSCs (Fig. 5B), suggesting that the hUC-MSCs on PLGA scaffold still maintained specific proteins of membranes and remained as multipotent. Taken together, the released hUC-MSCs from the PLGA@PLL scaffold could maintain their original properties, suggesting that the PLGA@PLL scaffold could serve as a perfect candidate system for hUC-MSCs culturing and production at a large scale.

## Discussion

Three-dimensional (3D) cell culture technology is a simple and effective method that is developed based on the two-dimensional (2D) cell culturing system. By comparison, the 3D scaffold prepared by electrospinning technology can better simulate the in vivo microenvironment due to its interconnectedness (Culenova et al. 2019; Zhao et al. 2022). The advantages of high porosity, ultra-high specific surface area, and easy-to-control preparation conditions of these 3D scaffold provide a simpler and more reliable research method for cell expansion in vitro. Currently, commonly used substrates include natural materials and synthetic polymer materials. Among them, a synthetic polymer material, such as PLGA, is safe, has good histocompatibility and plasticity, and can be processed into ideal structural shapes required for a variety of experiments (Raghav et al. 2022; Oller et al. 2015; Lanao et al. 2013).

By adopting these advantages, we developed a PLGA nanofiber scaffold coated with polylysine (PLGA@PLL) for large production of umbilical cord-derived mesenchymal stem cells (hUC-MSCs). The whole scaffold possessed an ideal pore size to incorporate the hUC-MSCs and the stable physicochemical properties of the scaffold allow it to act as a prolific, sustainable substrate for hUC-MSCs culture in vitro.

Usually, MSCs are cultured in a common culture dish in vitro*.* Once the growing cells come into contact with each other, movement, division, and proliferation are inhibited, resulting in a single layer of cells covering the bottom of the culture dish (Ribatti. 2017). Fortunately, the 3D scaffold prepared by electrospinning technology in this study can allow cells to grow in multiple directions along the fiber to utilize the spatial structure, which can greatly reduce the “contact inhibition” effect for cell proliferation.

In this study, unlike cells grow in the shape of a long spindle in the ordinary culture dish, we can observe that MSCs grow in the form of clumps on the 3D scaffold. More importantly, much more MSCs could be harvested from the PLGA@PLL scaffold compared with that from the unmodified scaffold and the culture dish (cell number of PLGA@PLL scaffold triple that of the culture dish, Fig. 2). The higher yield of hUC- MSCs possibly attributed to the PLGA@PLL scaffold afforded cells to grow in multiple directions along the nanofiber and to fully utilize the spatial structure, which can greatly decrease the “contact inhibition” effect for cell proliferation. It is worth noting that the expression of 2 genes which encode the key cellular proliferation biomarkers, including Ki67 and PCNA, and the other 2 genes which encode the key cellular adhesion biomarkers, including VCAM-1 and ICAM-1, were all found to be increased substantially in the hUC-MSCs derived from the PLGA@PLL scaffold compared to culture dish. The ensuing high expression of Ki67 and PCNA proteins could promote the proliferation of hUC-MSCs on the PLGA@PLL scaffold, while high expression of the cellular adhesion biomarkers could facilitate the cells adhere to the multiple levels of the PLGA@PLL scaffold which were beneficial for the cells to proliferate, thus lead to high yields of hUC-MSCs on this scaffold.

The results of the β-galactosidase staining analysis, pluripotent differentiation potential, and flow cytometry analysis showed no obvious difference between the native hUC-MSCs obtained from Wharton’s jelly and hUC-MSCs released from the PLGA@PLL scaffold. Furthermore, real-time PCR results showed that the cells growing on PLGA@PLL scaffold expressed much lower levels of P16 and P21 than that of the culture dish. These evidences demonstrate that PLGA@PLL scaffold could provide a better microenvironment for the expansion of hUC-MSCs. Faster cellular proliferation on the scaffold would result in faster cellular division and frequent cell cycles, which could generate more “young” offspring cells. Therefore, we propose that hUC-MSCs cultured by this 3D biomimetic substrate should have huge advantages over the traditional culturing patterns in terms of cellular proliferation, senescence, and maintaining multipotency and MSC stemness which could be ideally applied to downstream experiments and even clinical trials.

## Conclusions

In this study, we have successfully synthesized the PLL-grafted PLGA nanofibrous scaffold by electrospinning technology, which served as a good substrate for hUC-MSCs proliferation in vitro. This 3D scaffold had several advantages, such as good biocompatibility, non-toxicity, and easy preparation. In particular, after modification with PLL, the scaffold exhibited a better affinity toward hUC-MSCs than an unmodified one. Moreover, hUC-MSCs entered exponential growth earlier on this scaffold, resulting in a higher cell proliferation rate than in the culture dish. Furthermore, the hUC-MSCs remained intact in morphology, less senescent, and maintained the MSCs multipotency and stemness properties on the PLGA@PLL scaffold. All in all, this method could produce a large number of hUC-MSCs in a short time compared to the traditional method. Benefiting from this pattern, we hope this approach can serve as a promising and valuable platform for MSCs’ culture and application.

## Supplementary Information

**Additional file 1: Fig. S1.** Porosity of PLGA@PLL scaffold. Distributions of the size of 100 pores in SEM images of the 3D scaffold. **Fig. S2.** Degradation profile of the PLGA@PLL scaffold. The changes in dry weight of (A) PLGA and (B) PLGA@PLL scaffolds incubated in the culture medium over a period of 4 weeks. The dry weights of the scaffolds are presented as the mean ± SEM (n=5). (C) The changes in pH value of the culture medium incubated with PLGA@PLL scaffolds over a period of 4 weeks were measured and are presented as the mean ± SEM (n=5). *ns* indicated not significant (*p* > 0.05, one-way ANOVA tests). **Fig. S3.** hUC-MSCs isolated from the human umbilical cord were cultured on a 10 cm dish and observed under a microscope. **Fig. S4.** Pictures of newly prepared PLGA scaffolds (A) before and (B) after PLL modification were captured using a Nikon camera. **Fig. S5.** SEM images of hUC-MSCs seeded on the (A) PLGA scaffold and (B) PLGA@PLL scaffold. **Table S1.** Primers used in this study.

## Data Availability

All data supporting this article’s conclusion are available from the corresponding author on reasonable request.
