# Supplementary material for: Large-scale expansion of human umbilical cord-derived mesenchymal stem cells using PLGA@PLL scaffold
Source: Bioresour Bioprocess. 2023 Mar 8;10(1):18. doi: 10.1186/s40643-023-00635-6 (PMC9994782; doi:10.1186/s40643-023-00635-6)
Supplement: Supplementary file 1 — Additional file 1: Fig. S1. Porosity of PLGA@PLL scaffold. Distributions of the size of 100 pores in SEM images of the 3D scaffold. Fig. S2. Degradation profile of the PLGA@PLL scaffold. The changes in dry weight of (A) PLGA and (B) PLGA@PLL scaffolds incubated in the culture medium over a period of 4 weeks. The dry weights of the scaffolds are presented as the mean ± SEM (n=5). (C) The changes in pH value of the culture medium incubated with PLGA@PLL scaffolds over a period of 4 weeks were measured and are presented as the mean ± SEM (n=5). ns indicated not significant (p > 0.05, one-way ANOVA tests). Fig. S3. hUC-MSCs isolated from the human umbilical cord were cultured on a 10 cm dish and observed under a microscope. Fig. S4. Pictures of newly prepared PLGA scaffolds (A) before and (B) after PLL modification were captured using a Nikon camera. Fig. S5. SEM images of hUC-MSCs seeded on the (A) PLGA scaffold and (B) PLGA@PLL scaffold. Table S1. Primers used in this study. [file 40643_2023_635_MOESM1_ESM.docx]

Additional file for

**Large-scale expansion of human umbilical cord derived-mesenchymal stem cells using PLGA@PLL nanofiber scaffold**

Yujie Liu^1, 2^, Obed Boadi Amissah^1, 3^, Xiaoying Huangfang^4^, Ling Wang^5^, Habimana Jean de Dieu^1, 3^, Linshuang Lv^1, 3^, Xuanyan Ding^1, 3^, Junyi Li^1, 3^, Ming Chen^5^, Jinmin Zhu^6^, Omar Mukama^1^, Yirong Sun^1^, Zhiyuan Li^1, 3, 5-8,^ * and Rongqi Huang^1,^ ^7,^ *


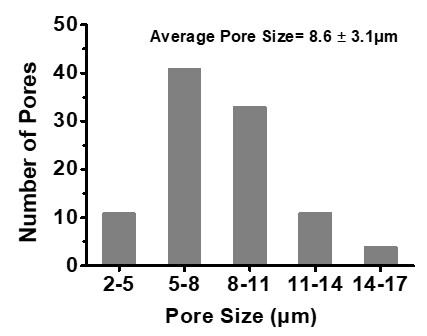


**Fig. S1** Porosity of PLGA@PLL scaffold. Distributions of the size of 100 pores in SEM images of the 3D scaffold.


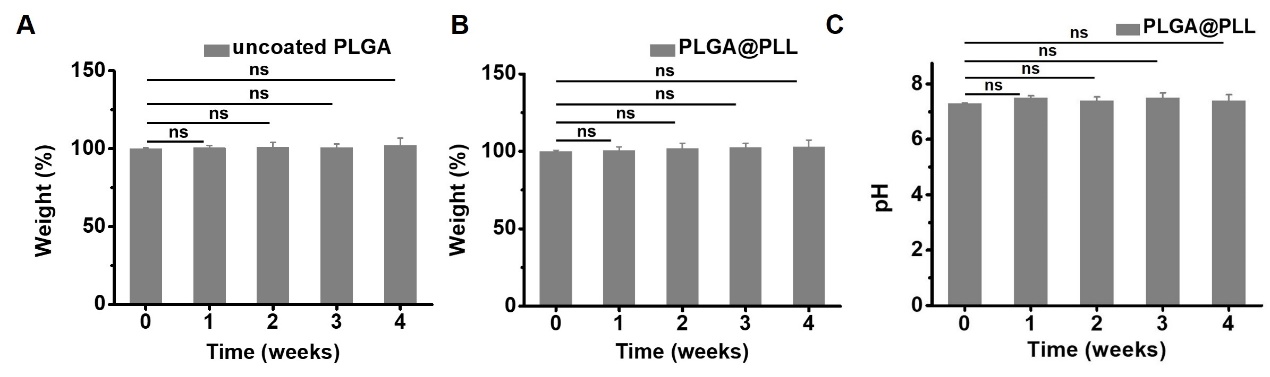


**Fig. S2** Degradation profile of the PLGA@PLL scaffold. The changes in dry weight of (A) PLGA and (B) PLGA@PLL scaffolds incubated in the culture medium over a period of 4 weeks. The dry weights of the scaffolds were presented as the mean ± SEM (n=5). (C) The changes in pH value of the culture medium incubated with PLGA@PLL scaffolds over a period of 4 weeks were measured and presented as the mean ± SEM (n=5). *ns* indicated not significant (*p* > 0.05, one-way ANOVA tests).


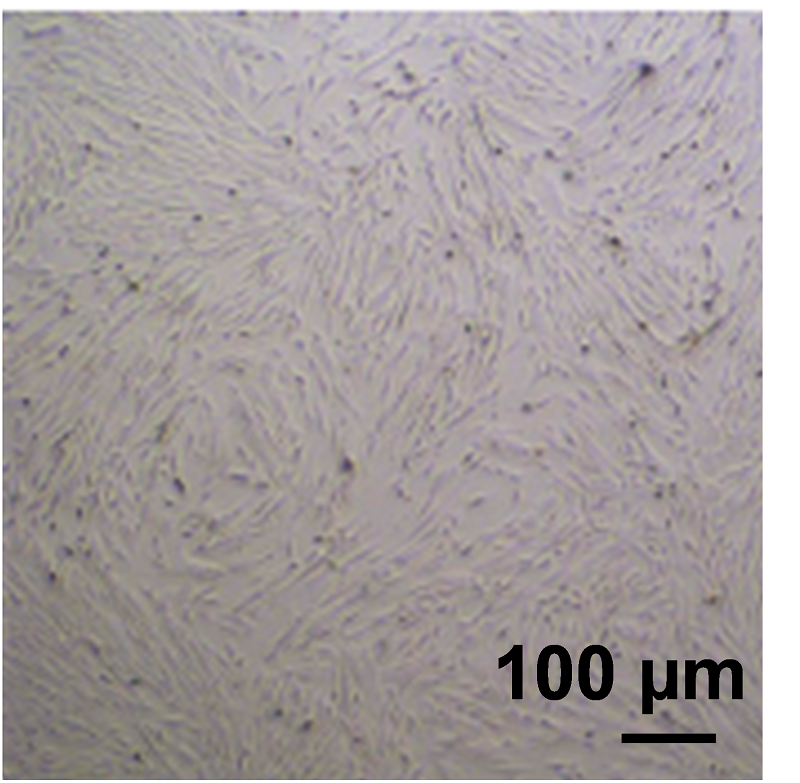


**Fig. S3** hUC-MSCs isolated from the human umbilical cord were cultured on a 10 cm dish and observed under a microscope.

**
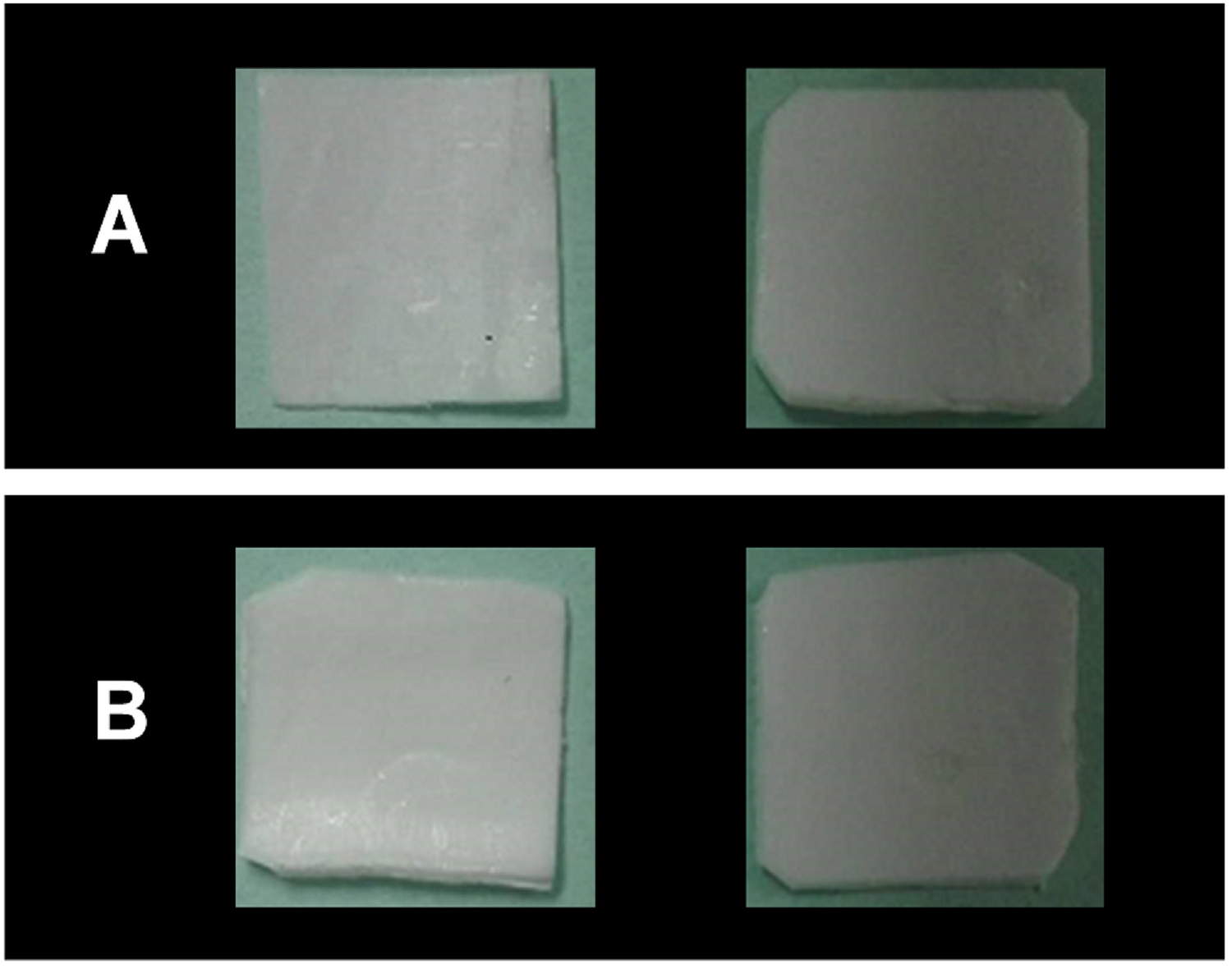
**

**Fig. S4** Pictures of newly-prepared PLGA scaffolds (A) before and (B) after PLL modification were captured using a Nikon camera.


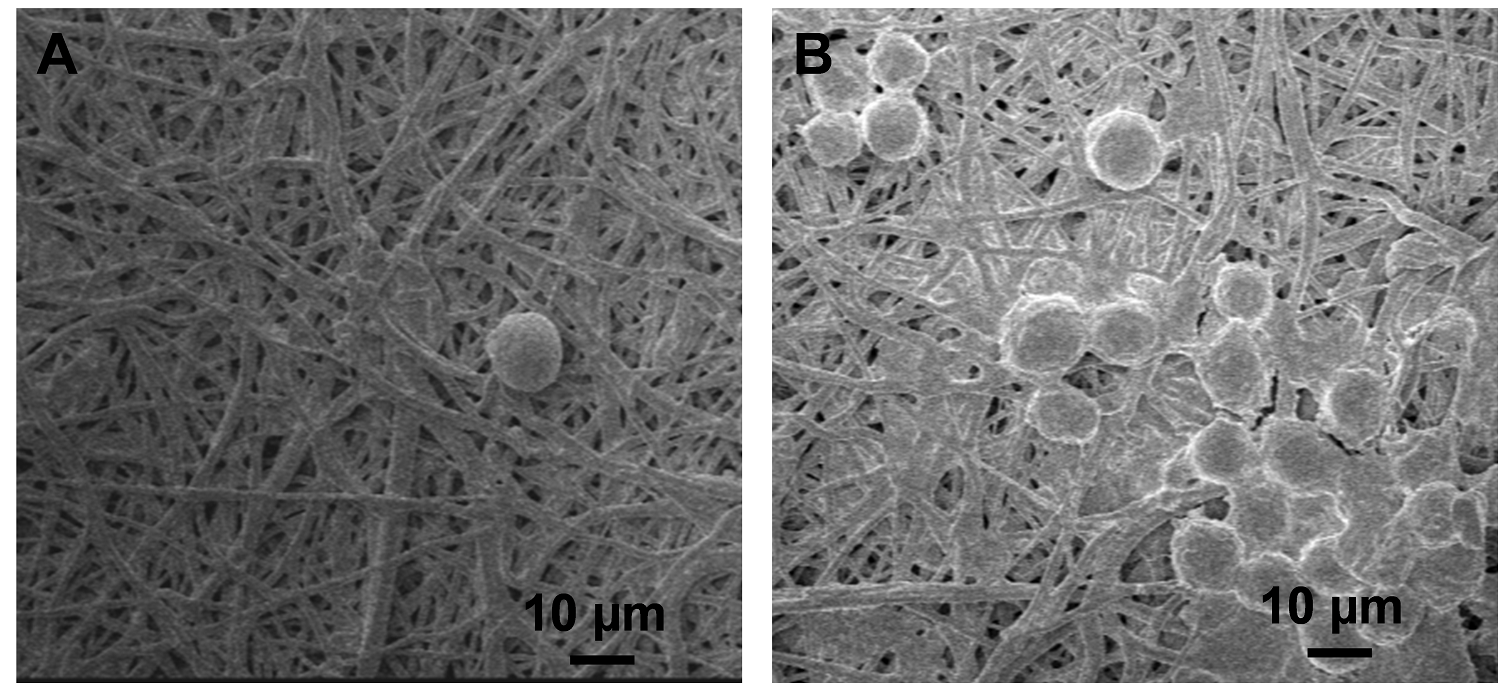


**Fig. S5** SEM images of hUC-MSCs seeded on the (A) PLGA scaffold and (B) PLGA@PLL scaffold.

**Table S1.** Primers used in this study

| **Primer** | **Sequence (5' to 3')** |
| --- | --- |
| Ki67  Ki67-F  Ki67-R | GAAAGAGTGGCAACCTGCCTTC  GCACCAAGTTTTACTACATCTGCC |
| PCNA  PCNA-F  PCNA-R | CAAGTAATGTCGATAAAGAGGAGG  GTGTCACCGTTGAAGAGAGTGG |
| ICAM-1  ICAM-1-F  ICAM-1-R | AGCGGCTGACGTGTGCAGTAAT  TCTGAGACCTCTGGCTTCGTCA |
| VCAM-1  VCAM-1-F  VCAM-1-R | GATTCTGTGCCCACAGTAAGGC  TGGTCACAGAGCCACCTTCTTG |
| P21  P21-F  P21-R | AGGTGGACCTGGAGACTCTCAG  TCCTCTTGGAGAAGATCAGCCG |
| P16  P16-F  P16-R | CTCGTGCTGATGCTACTGAGGA  GGTCGGCGCAGTTGGGCTCC |
